# Methyl Salicylate Lactoside Protects Neurons Ameliorating Cognitive Disorder Through Inhibiting Amyloid Beta-Induced Neuroinflammatory Response in Alzheimer’s Disease

**DOI:** 10.3389/fnagi.2018.00085

**Published:** 2018-03-27

**Authors:** Jinze Li, Xiaowei Ma, Yu Wang, Chengjuan Chen, Min Hu, Linlin Wang, Junmin Fu, Gaona Shi, Dongming Zhang, Tiantai Zhang

**Affiliations:** ^1^State Key Laboratory of Bioactive Substances and Functions of Natural Medicines, Department of Pharmacology, Institute of Materia Medica, Chinese Academy of Medical Sciences & Peking Union Medical College, Beijing, China; ^2^Department of Pharmacy, Beijing Shijitan Hospital, Capital Medical University, Beijing, China

**Keywords:** Alzheimer’s disease, cyclooxygenase, methyl salicylate lactoside, mitogen-activated protein kinase, neuroinflammation, neuron

## Abstract

Neuroinflammatory reactions mediated by microglia and astrocytes have been shown to play a key role in early progression of Alzheimer’s disease (AD). Increased evidences have demonstrated that neurons exacerbate local inflammatory reactions by producing inflammatory mediators and act as an important participant in the pathogenesis of AD. Methyl salicylate lactoside (MSL) is an isolated natural product that is part of a class of novel non-steroidal anti-inflammatory drugs (NSAID). In our previous studies, we demonstrated that MSL exhibited therapeutic effects on arthritis-induced mice and suppressed the activation of glial cells. In the current study, we investigated the effects of MSL on cognitive function and neuronal protection induced by amyloid-beta peptides (Aβ) and explored potential underlying mechanisms involved. Amyloid precursor protein (APP) and presenilin 1 (PS1) double transgenic mice were used to evaluate the effects of MSL through behavioral testing and neuronal degenerative changes. In addition, copper-injured APP Swedish mutation overexpressing SH-SY5Y cells were used to determine the transduction of cyclooxygenase (COX) and mitogen-activated protein kinase (MAPK) pathways. Our results indicated that at an early stage, MSL treatment ameliorated cognitive impairment and neurodegeneration in APP/PS1 mice. Moreover, in an *in vitro* AD model, MSL treatment protected injured cells by increasing cell viability, improving mitochondrial dysfunction, and decreasing oxidative damage. In addition, MSL inhibited the phosphorylated level of c-Jun N-terminal kinase (JNK) and p38 MAPK, and suppressed the expression of COX-1/2. As a novel NSAIDs and used for the treatment in early stage of AD, MSL clearly demonstrated cognitive preservation by protecting neurons via a pleiotropic anti-inflammatory effect in the context of AD-associated deficits. Therefore, early treatment of anti-inflammatory therapy may be an effective strategy for treating AD.

## Introduction

Alzheimer’s disease (AD) is the most common cause of dementia and is characterized by a decline in memory, language, problem-solving and other cognitive skills that affect a person’s ability to perform everyday activities (Alzheimer’s Association, 2016). It is well known that AD is an age-related neurodegenerative brain disorder that is characterized by the accumulation of amyloid-β peptides (Aβ), abnormally phosphorylated tau protein, and loss of neurons in the neocortex and hippocampus (Akiyama et al., 2000). In addition to the brain, Aβ is also known to be produced in peripheral tissues, and is secreted into the circulation. It can enter brain tissue through the blood brain barrier and contribute to AD-type pathological progression of the brain (Bu et al., 2018). In addition to these common characteristics, it is hypothesized that neuroinflammation plays an important role in AD development. Both innate immune and inflammatory reactions have been observed in the brain of AD patients (Wyss-Coray and Mucke, 2002). Aβ deposits induce local inflammatory reactions in the surrounding neurons marked by the production of various complementary components of CIq, C3 and C5 (Rogers et al., 1992; Heneka and O’Banion, 2007). These complementary factors further contribute to activation of microglia and astrocytes, thereby resulting in the release of multiple pro-inflammatory factors, including TNF-α, IL-1β, and IL-6, chemokines, cyclooxygenase-2 (COX-2) and reactive oxygen species (ROS; Akiyama et al., 2000). In previous studies, it was suggested that these factors contributed to neuronal dysfunction and cell death, and the progression of dementia (Abbas et al., 2002; Brown and Bal-Price, 2003). In addition, mast cells play a crucial role in neuroinflammatory reaction in AD progression (Kempuraj et al., 2017). Mast cells contain a vast amount of pro-inflammatory mediators stored in secretory granules that, upon activation, may quickly release their contents into the extracellular environment. Moreover, mast cells are highly sensitive to Aβ protein activation. Degranulation responses of activated mast cells may lead to extracellular release of pro-inflammatory mediators, resulting in neuroinflammatory activities that promote AD progression (Harcha et al., 2015).

Several clinically-oriented epidemiological studies have indicated a lower incidence and progression of AD in arthritis patients who received non-steroidal anti-inflammatory drugs (NSAIDs; McGeer et al., 1990; Akiyama et al., 2000). Some of these beneficial effects have been confirmed in both *in vitro* assays and transgenic AD mice and demonstrated that treatment with celecoxib, ibuprofen, indomethacin, or naproxen reduced the production of pro-inflammatory mediators initiated by microglial and astrocyte activation, as well as by the deposition of Aβ_42_ (Weggen et al., 2001; Sastre et al., 2006; Mhillaj et al., 2018). However, other clinical trials using selective COX-2 inhibitors have failed to improve the behavior and cognitive skills in AD patients (Thal et al., 2005). Based on the above-mentioned paradoxical or questionable data, the efficacy of anti-inflammatory therapy needs to be further confirmed in AD.

Although the therapeutic effects of NASIDs are contradictory, the inflammatory responses are unquestionable accompanying the disease development in AD. To date, the underlying mechanisms of NSAIDs in preventing AD are unclear, and several inflammatory-related signal pathways, such as NF-κB and mitogen-activated protein kinase (MAPK) pathways, have shown to play a key role in neuroinflammatory responses in the brain (Yu et al., 2015). P38 MAPK and c-Jun N-terminal kinase (JNK) belong to the MAPK family and are responsive to stress stimuli, such as inflammatory cytokines and ROS. Activation of microglia-associated p38 MAPK and the downstream-related overproduction of pro-inflammatory cytokines lead to loss of synaptic proteins, neurite degeneration, and neuronal death in co-cultures of microglia and neurons. Reduction of p38 MAPK expression in neurons exerted neuroprotective effects and attenuated the generation of Aβ in mice (Xing et al., 2014; Schnöder et al., 2016). Therefore, targeting neuroinflammatory regulation may be a valuable therapeutic strategy in patients with AD.

Increasing evidence has shown that natural products, such as curcumin (Morales et al., 2017) and triptolide (Cui et al., 2016), play a key role in the anti-inflammatory effect of neurodegenerative AD. In our previous study, we demonstrated that methyl salicylate lactoside (MSL), a novel NASIDs derived from a natural product, *Gaultheria yunnanensis (Franch.) Rehder*, exerted significant anti-neuroinflammatory effects by inhibition of the activation of NF-κB pathway and COX in a non-selective manner in primary glial cells (Lan et al., 2011). However, no literature is available on the effects of MSL in AD animal models or on neuronal cells. Moreover, in a previous study it was demonstrated that MSL exerted therapeutic effects on collagen-induced arthritis mice by suppressing inflammatory responses without damaging gastric mucosa (Xin et al., 2014). Therefore, MSL may be a potential therapeutic agent that regulates inflammatory disorders. Based on the findings presented in an epidemiological study of NSAIDs (Wyss-Coray and Rogers, 2012), we investigated whether NSAIDs could prevent AD development.

The aim of the present study was to determine whether MSL prevented early progression of dementia in AD-related amyloid precursor protein (APP)/presenilin 1 (PS1) double transgenic mice, and to explore the potential mechanisms of action involved in neuronal protection. Our results indicated that MSL ameliorated cognitive disorders in an AD-related transgenic mouse model by inhibiting MAPK pathway activation in neuronal cells.

## Materials and Methods

### Reagents

MSL (99% purity) was synthesized by the Department Natural Medical Chemistry, Institute of Materia Medica, Chinese Academy of Medical Sciences & Peking Union Medical College (Beijing, China). All chemicals were purchased from Sigma-Aldrich (St. Louis, MO, USA), unless otherwise specified. Cell culture reagents were purchased from Invitrogen Corporation (Thermo Fisher Scientific, Carlsbad, CA, USA).

### Animals and Treatment

Heterozygous APPswe/PS1Δ9 transgenic founder mice (males and females, 8 weeks of age) were used as an AD model, which express mutant APP and PS1 under the control of endogenous promoters and show AD pathology without APP or PS1 overproduction. Age-matched wild-type (WT) littermates were used as controls. Mice, aged 8 weeks and weighting 20 ± 2 g, were housed five per cage at a constant temperature of 22 ± 4°C, humidity 60%–65%. Mice were fed standard laboratory chow and water *ad libitum*, and were kept under a 12 h dark/light cycle. The experimental procedures were approved by the Experimental Animal Care and Use Committee of the Institute of Materia Medica, Chinese Academy of Medical Sciences & Peking Union Medical College. Studies involving animals were carried out in accordance with the recommendations of the Council for International Organization of Medical Sciences on Animal Experimentation (World Health Organization, Geneva, Switzerland).

APP/PS1 mice were randomly divided into one model group and two treatment groups, whereas WT mice served as the control group (*n* = 12 per group). For all treatment groups, mice were given MSL (150 mg/kg and 300 mg/kg, dissolved in 0.5% sodium carboxyl methyl cellulose, CMC-Na, respectively) by p.o. once a day for 4 months. In addition, APP/PS1 and WT mice were administered CMC-Na vehicle p.o. using the same administration paradigm as controls. After completion of behavioral testing, half of the mice per group were sacrificed by an intraperitoneal overdose of chloral hydrate. Brains were quickly dissected and brain tissue was snap frozen in liquid nitrogen, and then stored at −80°C for further analysis. The remaining mice in each group were anesthetized and perfused from the left ventricle with 0.9% saline (Beijing Chemical Works, Beijing, China), followed by 4% paraformaldehyde (PFA, pH 7.40–7.50; Beijing Chemical Works, Beijing, China) to perfuse and fix brain tissue. Next, brains were dissected, embedded in paraffin, and stored at room temperature for histopathological analysis.

### Morris Water Maze Test

The Morris water maze (MWM) test was performed to evaluate the effect of MSL on learning and memory ability as previously described (Liu et al., 2012). In brief, the test involved five consecutive days of training trials, followed by one probe trial on the 6th day. Mice were released in a fixed direction of the maze and the time required to escape onto a hidden platform was recorded as escape latency. Mice were given a maximum of 60 s to find the hidden platform. For the probe trial, the number times that mice remained in the platform quadrant as well as the number of times that mice crossed through the platform quadrant during the 60 s-period were recorded. Treatments continued during the MWM test.

### Transmission Electron Microscope Analysis

Transmission electron microscope (TEM) was performed to estimate the level of neuronal degeneration in brain tissue as previous described (Liu et al., 2012). In brief, mice were anesthetized and perfused with 0.9% saline and 4% PFA, respectively. Brains were rapidly removed and the temporal cerebral cortices were carefully isolated and placed overnight in fixative (2.5% glutaraldehyde, Merck, Darmstadt, Germany). Then, samples were post-fixed in 1% osmium tetroxide, stained in 2% uranyl acetate, dehydrated in ethanol (Beijing Chemical Works, Beijing, China) and acetone (Beijing Chemical Works, Beijing, China), and embedded in epoxy resin (Beijing Zhongjingkeyi Technology, Beijing, China). Ultrathin sections (60 nm thick) of the samples were mounted on copper grids (200 mesh; Beijing Zhongjingkeyi Technology, Beijing, China) and double-contrasted with uranyl acetate and lead citrate for visualization using a TEM (Hitachi H-7650, Japan), which was operated at 80 kV.

### Fluoro-Jade B Staining Assay

The brain cerebral cortex of APP/PS1 mice was assessed for neuronal degeneration by the Fluoro-Jade B (FJB) assay as described previously (Schmued and Hopkins, 2000). In brief, brain tissues were paraffin embedded, sectioned (4 μm), mounted on slides, and dried overnight at room temperature. Next, sections were immersed in a solution, containing 1% sodium hydroxide in 80% ethanol for 5 min, followed by 2 min in 70% ethanol, and 2 min in distilled water. Sections were then transferred to a solution containing 0.06% potassium permanganate for 10 min, rinsed in distilled water for 2 min, and transferred to a 0.0004% FJB staining solution (Histochem, Jefferson, AR, USA) for 20 min. After washing with triple distilled water, the sections were placed in a warmer at 50°C until they were fully dry. The dry sections were cleared by immersion in xylene and cover-slipped with mounting medium (Sigma Chem Co., St. Louis MO, USA) for histological analysis. Staining intensity was visualized using a fluorescent microscope (Olympus IX71, Tokyo, Japan) using blue excitation light and a barrier filter. Degenerated neurons are stained with FJB and show a bright fluorescence intensity, indicating FJB-positive neurons, when compared to the background. The number of FJB-positive neurons was calibrated as the number of neurons in 1 mm^2^ of the cerebral cortex region. Cell counts were obtained by averaging the counts from 10 sections per mouse.

### Cell Culture and Treatment

Human neuroblastoma SH-SY5Y cells, which overexpressing the Swedish mutant form of human APP (referred to as “APPsw cells”) as an AD cell model by using copper to trigger the neurotoxicity of Aβ, were used to evaluate the effect of MSL. In brief, SH-SY5Y cells were cultivated in DMEM/F12 medium supplemented with 10% fetal bovine serum (FBS) and maintained under standard conditions of 37°C, 5% CO_2_, and 95% humidity. For immunofluorescence analysis, cells were seeded at a density of 8000 cells per well in black-walled optically clear-bottomed 96-well plates (Corning Life Sciences, Acton, MA, USA). Cells were treated with MSL (50 μM, 100 μM and 200 μM) and copper (250 μM) and incubated for 12 h. Then, cells were collected to evaluate the effects of  MSL.

### Cell Viability Assay

Cell viability was determined by a 3-(4, 5-dimethylthia-zol-2-yl)-5- (3-carboxymethoxyphenyl)-2-(4-sulfophenyl)-2H-tetrazolium (MTS) assay (Promega, Madison, WI, USA) according to the manufacturer’s guidelines. In brief, cells were deprived of serum for 12 h, treated with MSL (0–200 μM) for an additional 48 h, then incubated in MTS solution for another 1 h. Next, the absorbance (490 nm) was measured using a SpectraMax Plus microplate reader (Molecular Devices Corp., Sunnyvale, CA, USA).

### Mitochondrial Membrane Potential and Superoxide Detection

The mitochondrial membrane potential (MMP) and superoxide levels of APPsw cells were monitored by using the fluorescent dye of Rhodamine 123 (Rh123) (Dojindo Laboratory, Kumamoto, Japan) and MitoSOX Red (Invitrogen, Carlsbad, CA, USA), respectively. Depolarization of the MMP will cause Rh123 to be permeated from the mitochondrion into the intercellular space (Satoh et al., 1997). MitoSOX Red is a special mitochondrial superoxide indicator that targets to mitochondria and shows red fluorescent when it is oxidized by superoxide (Hu et al., 2007). After treatment of the APPsw by MSL and copper as described above, Rh123 and MitoSOX Red at final doses of 10 μM and 5 μM, respectively, were added to cells for 30 min. Subsequently, 10 min before the end of the staining, 10 μM of the nucleic acid dye Hoechst 33342 (Dojindo Laboratory, Kumamoto, Japan) was added to the culture to locate the nucleus. Fluorescent images and intensities were acquired and analyzed by a Cellomics ArrayScan VTI HCS Reader (Thermo Fisher Scientific Cellomics, Pittsburgh, PA, USA) that was combined with a Cell Health Profiling BioApplication Guide provided with the BioApplication software. Values of target mean average fluorescent intensities (Mean_TargetAvgInten) were recorded as experimental data.

### Measurements of Intracellular ROS

The level of cytoplasmic ROS in APPsw cells was measured by using a CellROX^®^ Deep Red Reagent (Invitrogen, Carlsbad, CA, USA), which was designed to reliably measure reactive ROS in live cells. In brief, the cell-permanent dye of this reagent is non-fluorescent when in a reduced state, and exhibits bright fluorescence upon oxidation by ROS. Culturing of APPsw and treatment with MSL or copper was as described above. ROS measurement was performed according to the manufacturer’s instructions. Fluorescence intensity was determined and quantified as described before using the Cellomics ArrayScan VTI HCS Reader. Values of target mean average fluorescent intensities (Mean_TargetAvgInten) were recorded as experimental data.

### Determination of COX Activation and MAPK Signal Pathways

COX expression, prostaglandin E2 (PGE_2_) production, MAPK signaling pathway, and TNF-α content were assessed by immunofluorescence assay to determine the effect of MSL treatment on APPsw cells. After treatment with MSL and copper as described above, APPsw cells were fixed with 4% PFA, permeabilized with 0.3% Triton X-100, and blocked with 3% bovine serum albumin (BSA) at room temperature. Next, a primary antibody mixture, containing anti-COX-1 (1:200, Abcam, Cambridge, MA, USA), anti-COX-2 (1:200, Abcam, Cambridge, MA, USA), anti-PGE_2_ (1:200, Abcam, Cambridge, MA, USA), anti-p-p38 MAPK (1:200, Abcam, Cambridge, MA, USA), anti-p-JNK (1:200, Abcam, Cambridge, MA, USA), anti-p-ERK1/2 (1:200, Abcam, Cambridge, MA, USA), and anti-TNF-α (1:400, Cell Signaling Technology, Beverly, MA, USA) in DMEM/F12 culture medium was added to the cells and incubated for 2 h at room temperature. Subsequently, cells were incubated with corresponding-matched Alexa Fluor 546-conjugated goat anti-rabbit/mouse secondary antibodies (1:2000, Invitrogen, Carlsbad, CA, USA) for 1 h. Fluorescent images were acquired and intensity was quantified by a Cellomics ArrayScan VTI HCS Reader with a BioApplication Software Module (Thermo Fisher Scientific Cellomics, Pittsburgh, PA, USA). The content of intracellular proteins was represented by the average fluorescence intensity (Mean_TargetAvgInten). In addition, differences between the intensity of the nuclear and cytoplasmic fraction (Mean_CircRingAvgIntenDiff) were acquired and calculated as the extent of protein translocation.

### Statistical Analysis

Data were presented as the mean ± SEM. Place navigation data of the MWM test were analyzed by two-factor analysis of variance (ANOVA) with repeated measures. Tukey’s *post hoc* test was used if the treatment was significant by ANOVA. Other data were analyzed using one-way ANOVA, followed by an appropriate *post hoc* test. *In vitro* assays were performed at least in triplicate. *p* < 0.05 was considered statistically significant.

## Results

### MSL Treatment Improves Cognitive Deficits in APP/PS1 Mice

**Figure 1:**
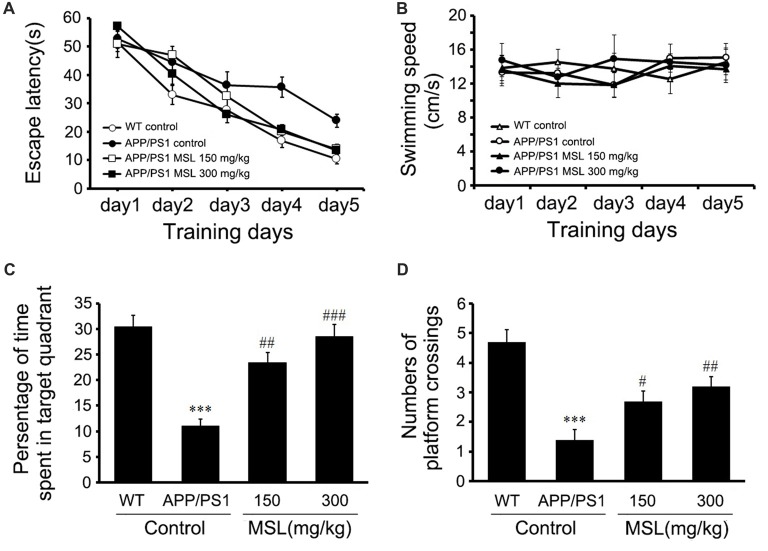
Methyl salicylate lactoside (MSL) improved learning and memory in amyloid precursor protein (APP)/presenilin 1 (PS1) mice. **(A)** Latency to reach the escape platform during five training days in the Morris water maze (MWM) test showed a significant effect on escape latency (*F*_(4,144)_ = 117.763, *p* < 0.001) between groups. **(B)** During the training period, no significant differences were observed in swimming speed of mice among groups. **(C)** In the probe test, MSL-treated mice showed a significant increase in the time spent in the target quadrant when compared to the APP/PS1 control mice. **(D)** In the probe test, MSL-treated mice showed an increase in the number of platform crossings when compared to APP/PS1 control mice. Data are presented as the mean ± SEM, *n* = 12 mice per group. ***p < 0.001 vs. wild-type (WT) control mice; ^#^*p* < 0.05, ^##^*p* < 0.01, ^###^*p* < 0.001 vs. APP/PS1 control mice.

To evaluate the effect of MSL treatment on Aβ-dependent behavioral changes in early Alzheimer-related impairments, spatial learning was assessed by the time required to find the hidden platform (escape latency) in the place navigation trial of the MWM test. Two-way ANOVA with repeated measurements illustrated a significant effect on escape latency (*F*_(4,144)_ = 117.763, *p* < 0.001, Figure 1A) between groups. These findings indicated that mice in different groups showed different spatial learning capabilities during the 5-day acquisition training. The results also demonstrated that mice treated with MSL (150 mg/kg, *p* < 0.05; 300 mg/kg, *p* < 0.05) demonstrated a significant reduction in latency time on day 4 and 5 when compared to the APP/PS1 control mice. In addition, our results indicated that the swimming speed of mice during the training period was not significantly different between groups (Figure 1B), suggesting that there were no sensorimotor abnormalities.

In the probe test, APP/PS1 control mice showed a significant decrease in the mean time spent in the target quadrant compared to WT mice (*p* < 0.001, Figures 1C,D). Moreover, treatment with MSL (150 mg/kg and 300 mg/kg) remarkably elongated the search time in the target quadrant (*p* < 0.01 and *p* < 0.001, Figure 1C) and increased platform crossing numbers of APP/PS1 mice (*p* < 0.05 and *p* < 0.01, Figure 1D) in a dose-dependent manner. These results suggested that MSL improved spatial learning and memory capability of aging mice.

### MSL Treatment Protected the Cerebral Cortex and Hippocampus Ultrastructure in APP/PS1 Mice

**Figure 2:**
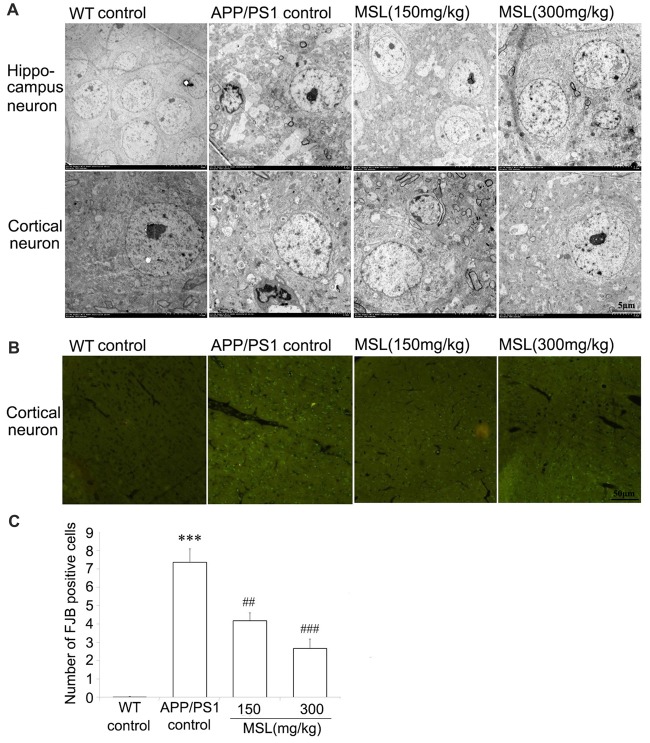
MSL protected ultrastructural neuronal damage and degeneration. **(A)** Ultrastructural analysis indicated that MSL treatment prevent rupture of the neuronal membrane and edema of neurons in the hippocampus or cortex. **(B)** Fluoro-Jade B (FJB) staining assay was used to evaluate the effects of MSL treatment on cortical neuronal degeneration. MSL treatment significantly prevented the generation of FJB-positive cells. **(C)** Mean number of FJB-positive cells/mm^2^ of the cerebral cortex. Data are presented as the mean ± SEM, *n* = 6 mice per group. ***p < 0.001 vs. WT control mice; ^##^*p* < 0.01, ^###^*p* < 0.001 vs. APP/PS1 control mice.

To further assess the changes in morphological ultrastructure of neurons, ultra-thin sections of brain tissue were evaluated under a TEM. Our results indicated that neurons in the cerebral cortex and hippocampus of WT mice showed a normal appearance (Figure 2A). Neither swelling nor shrinkage was observed and no signs of perivascular edema were present. However, in the brain of APP/PS1 mice, rupture of the neuronal membrane as well as edema of neurons were observed in the cortex and hippocampus. MSL treatment at doses of 150 mg/kg and 300 mg/kg markedly attenuated neuropil damage in the hippocampus and cortex. Neuronal pyknosis was relieved and signs of membrane damage and vacuolization were less apparent. After MSL treatment, the number of synaptic vesicles was restored and the length and thickness of synapses were improved.

### MSL Treatment Inhibited Neuronal Degeneration in the Cerebral Cortex

FJB staining identifies neurons that actively undergo degeneration in brain sections. Degenerated neurons were stained using a FJB immunofluorescence approach to evaluate neuronal activity in AD. As showed in Figure 2B, intense fluorescence, indicating FJB-positive cells, was highly present in the cerebral cortex of APP/PS1 but not WT mice. In MSL-treated APP/PS1 mice, intense fluorescence in the cerebral cortex was significantly alleviated in a dose-dependent manner, when compared to untreated APP/PS1 mice (Figure 2C). These results implied that MSL protected neuronal degeneration.

### MSL Increases Cell Viability

**Figure 3:**
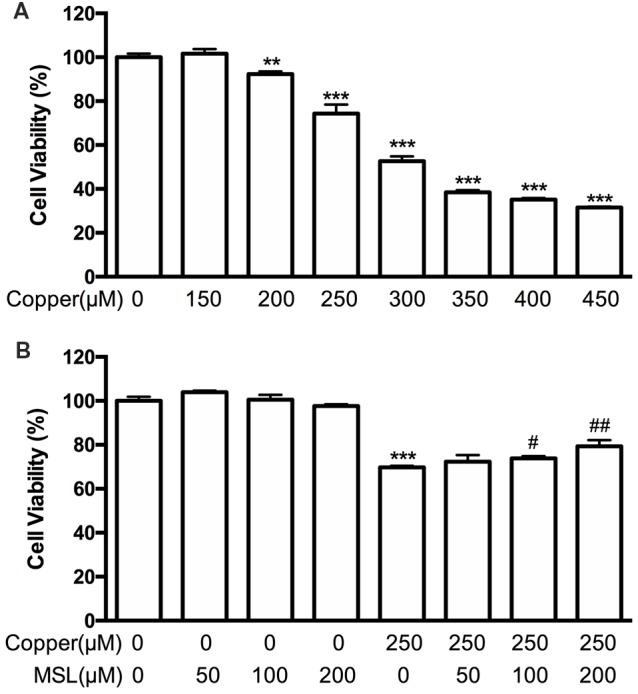
Effects of MSL treatment on the viability of APPsw cells in the presence of copper. **(A)** Cell viability was assessed by MTS assay in the presence of copper. **(B)** MSL prevented the damage of APPsw by copper. Data are expressed as the mean ± SEM from three independent experiments. **p < 0.01, ***p < 0.001 vs. untreated cells; ^#^*p* < 0.05, ^##^*p* < 0.01 vs. copper-treated cells.

Copper was used to induce neurotoxicity of Aβ in APPsw cells. First, we evaluated the viability of copper-treated APPsw cells. Our results indicated that administration of copper for 48 h significantly decreased cell viability in a concentration-dependent manner (Figure 3A). In subsequent *in vitro* assays, the concentration of copper was chosen as 250 μM. Treatment with MSL (50–200 μM) did not have any cytotoxic effect on APPsw cells. However, copper–induced cytotoxicity was reduced by MSL (Figure 3B). In the presence of copper, cell viability significantly decreased in a dose dependent manner. Our results indicated that MSL treatment converted copper-induced cell damage, and an MSL dose of 100 and 200 μM significantly prevented cellular damage in a concentration-dependent manner (100 μM, *p* < 0.05; 200 μM, *p* < 0.01). In addition, MSL did not have any cytotoxic effect in APPsw cells in the absence of copper.

### MSL Increases the Mitochondrial Membrane Potential of APPsw Cells in the Presence of Copper

**Figure 4:**
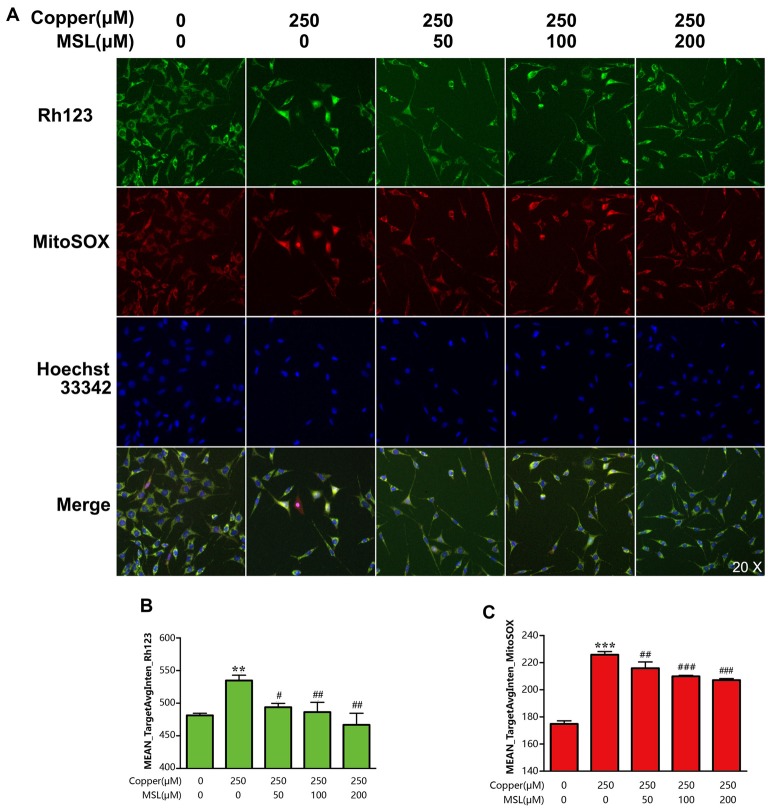
MSL alleviated mitochondrial dysfunction of copper-induced APPsw cells. **(A)** Mitochondrial ability was assessed by mitochondrial membrane potential (MMP) and superoxide levels. Fluorescent images of Rh123 and MitoSOX indicated the changes of MMP and superoxide, respectively. Fluorescent images and intensities were analyzed by a Cellomics ArrayScan high-content screening (HCS) Reader and software. Quantified values of Rh123 **(B)** and MitoSOX **(C)** were analyzed by Mean_Avginten. Data are expressed as the mean ± SEM, *n* = 6, **p < 0.01, ***p < 0.001 vs. untreated cells; ^#^*p* < 0.05, ^##^*p* < 0.01, ^###^*p* < 0.001 vs. copper-treated cells.

To evaluate the mitochondrial ability, the MMP of APPsw cells was determined by fluorescent analysis. After incubation with copper for 24 h, the Mean_TargetAvgInten values of Rh123 and MitoSOX increased to 64% (*p* < 0.01, Figures 4A,B) and 156% (*p* < 0.001, Figures 4A,C) compared to the control group, respectively. Treatment with Rh123 and MitoSOX showed a significant reduction in MMP as well as in the production of superoxide in mitochondria, respectively. However, MSL significantly relived mitochondrial dysfunction of copper-treated APPsw cells. The Mean_TargetAvgInten values of Rh123 (Figures 4A,B) and MitoSOX (Figures 4A,C) were reduced by MSL in a concentration-dependent manner.

### MSL Treatment Decreases the Generation of ROS in Appsw Cells

**Figure 5:**
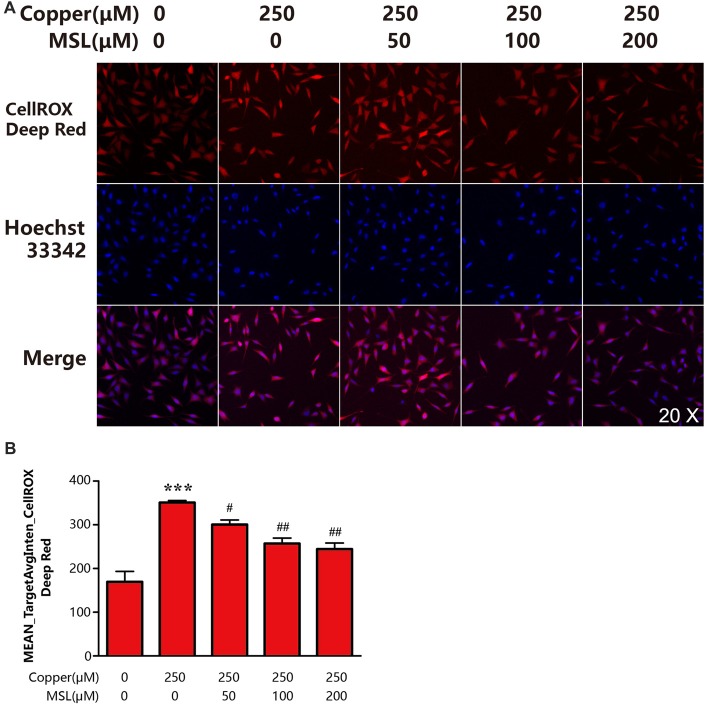
MSL inhibited the production of intracellular reactive oxygen species (ROS) in APPsw cells. **(A)** Fluorescent images of CellROX Deep Red representing ROS production. Fluorescent images and intensities were analyzed by a Cellomics ArrayScan high-content screening (HCS) Reader and software. **(B)** Quantified values of CellROX Deep Red was analyzed by Mean_Avginten. Data are expressed as the mean ± SEM, *n* = 6, ***p < 0.001 vs. untreated cells; ^#^*p* < 0.05, ^##^*p* < 0.01 vs. copper-treated cells.

To determine the state of oxidative stress of neuronal cells, the production of ROS by APPsw cells was determined. Our results indicated that copper induced significant oxidative stress levels or ROS production by a 1.0-fold increase when compared to control APPsw cells (Figures 5A,B). However, treatment with MSL significantly prevented the formation of ROS in copper-treated APPsw cells at each concentration tested (Figures 5A,B), indicating that MSL prevented the ability of APPsw cells to produce oxidants.

### MSL Suppresses COX1/2 Activation and the Expression Of PGE_2_ of Appsw Cells

**Figure 6:**
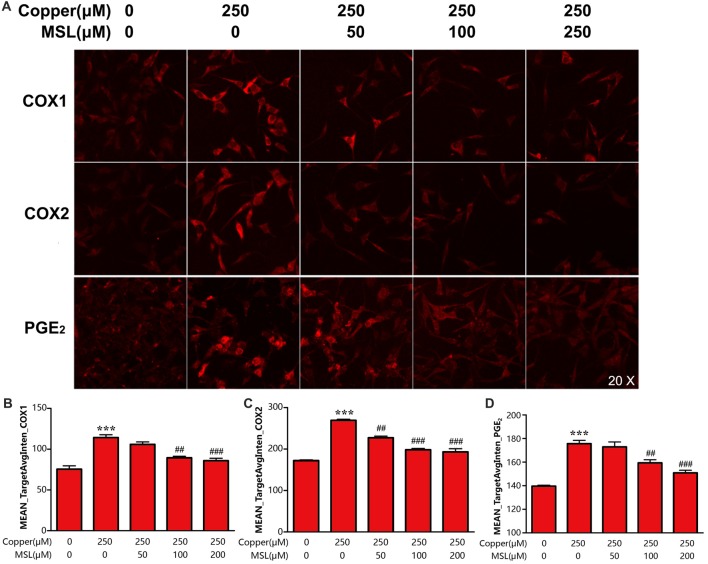
MSL inhibited the expression of cyclooxygenase (COX) and prostaglandin E2 (PGE2) generation of APPsw cell in the presence of copper. **(A)** Immunofluorescence analysis was used to determine the expression of COX1/2 and the production of PGE_2_ of APPsw cells in the presence of copper. Fluorescent images and intensities were analyzed by a Cellomics ArrayScan high-content screening (HCS) Reader and software. Quantified values of COX-1 **(B)**, COX-2 **(C)** and PGE_2_ **(D)** were analyzed by Mean_Avginten. Data are expressed as the mean ± SEM, *n* = 6, ***p < 0.001 vs. untreated cells; ^##^*p* < 0.01, ^###^*p* < 0.001 vs. copper-treated cells.

PGE_2_ is an important inflammatory mediator that is catalyzed and produced by the two isoforms of COXs. In the present study, we evaluated the effect of MSL treatment on the production of PGE_2_ and expression of COX-1/2 in APPsw cells. The results showed that APPsw cells expressed a high level of COX-1 and COX-2 after exposure to 250 μM of copper, which could be prevented by MSL in a concentration-dependent manner (Figures 6A–C). Moreover, MSL exhibited anti-neuroinflammation effects by suppressing the production of copper-induced PGE_2_ in APPsw cells (Figures 6A,D).

### MSL Prevents MAPK Pathways in APPsw Cells

**Figure 7:**
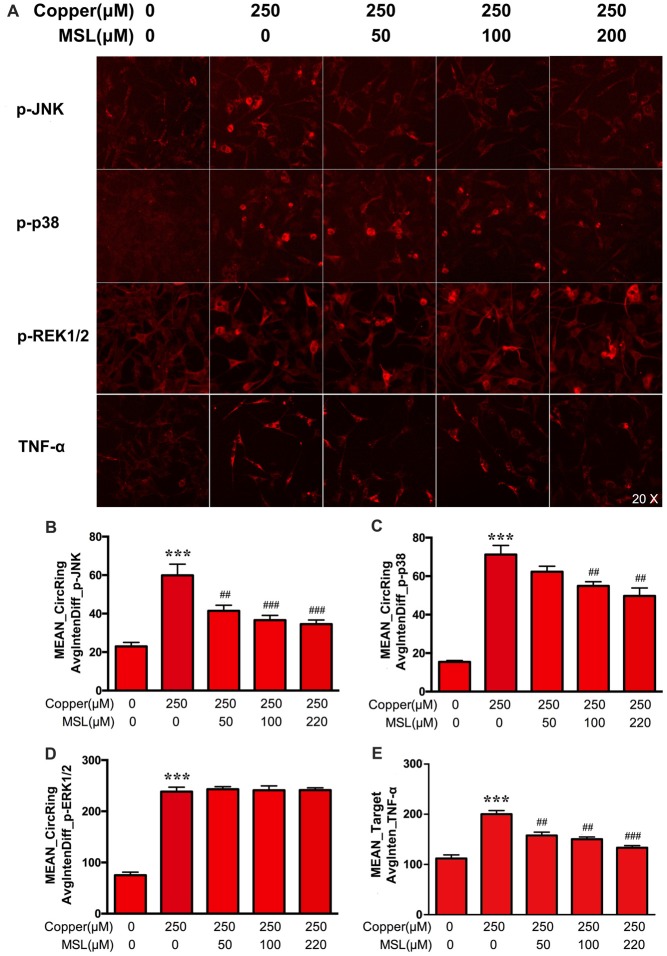
The effect of MSL on activation of the mitogen-activated protein kinase (MAPK) signal pathway in APPsw cells. **(A)** Immunofluorescence analysis was used to determine the phosphorylation of c-jun n-terminal kinase (JNK), p38, ERK1/2, and the production of TNF-α by APPsw cells in the presence of copper. Fluorescent images and intensities were analyzed by a Cellomics ArrayScan high-content screening (HCS) Reader and software. Quantified values of JNK **(B)**, p38 **(C)**, ERK1/2 **(D)** and TNF-α **(E)** were analyzed by Mean_Avginten. Data are expressed as the mean ± SEM, *n* = 6, ***p < 0.001 vs. untreated cells; ^##^*p* < 0.01, ^###^*p* < 0.001 vs. copper-treated cells.

MAPK pathways play a pivotal role in aberrant pro-inflammatory responses in neurodegenerative conditions in the central nervous system (Xing et al., 2014). Here, the phosphorylated expression of JNK, p38 and ERK1/2 of MAPKs was determined in APPsw cells to evaluate the treatment effect of MSL. As shown in Figures 7A–E, the expression of p-p38, p-JNK, and p-ERK1/2 was significantly increased in copper-induced APPsw cells. MSL pre-treatment prevented the increase in copper-induced phosphorylation of p38 (Figure 7B) and p-JNK (Figure 7C), in a concentration-dependent manner, but not p-ERK1/2 (Figure 7D). In addition, MSL pre-treatment significantly decreased copper-induced production of the pro-inflammatory cytokine TNF-α in APPsw cells (Figure 7E).

## Discussion

Previous studies have shown that amyloid-β damages neurons and induces neuroinflammatory activation in the brain (Wyss-Coray and Rogers, 2012). In the present study, we evaluated the effect of MSL, a novel NSAID derived from natural products, on neuronal protection against Aβ neurotoxicity in both transgenic APP/PS1 mice and APPsw cells in the presence of copper. Our results demonstrated for the first time that MSL treatment prevented the progression to cognitive deficits in APP/PS1 mice in the early stages of AD. In an *in vitro* model of AD, MSL protected neuronal cells by preventing the generation of ROS and by improving mitochondrial ability. Moreover, MSL treatment non-selectively inhibited the expression of COX-1 and COX-2, and subsequent PGE_2_ production. In neuronal cells, MSL treatment reduced copper-induced phosphorylation of p38 MAPK and JNK. These findings complemented and extended the findings presented in our previous study in which we demonstrated the anti-inflammatory effects of MSL by inhibiting activation of the NF-κB signaling pathway in primary glial cells (Lan et al., 2011).

Neuropathological studies in patients demonstrated that neuroinflammatory changes are relatively early events that were indicated by the activation of gliacyte and neurons, and increased neuronal expression of COX-2. These early stages of AD pathology preceding the stages that are associated with clinical dementia syndrome (Hoozemans et al., 2006; Parachikova et al., 2007). Epidemiological and genetic studies have indicated that inflammation-related mechanisms can contribute to the multifactorial etiology of the sporadic late-onset of AD (Eikelenboom et al., 2011). These results predicted that NSAIDs may be effective candidates for the treatment of AD. In addition, subsequent clinical studies have shown that therapeutic approaches targeting inflammatory processes were an effective strategy for slowing down disease progression and dramatically decreased disease incidence and risk (Rogers et al., 1993; McGeer and McGeer, 1996). However, in other studies it was shown that several clinical trials, in which NSAIDs were used for treating AD patients, failed in preventing the progression or improvement of cognitive disorders of AD (Green et al., 2009; Wyss-Coray and Rogers, 2012). When analyzing these clinical trials, it is obvious that they lack large-scale clinical multicenter trials to treat AD using NSAIDs. In addition, these trials were short, used a sub-therapeutic dose, were different in selectivity and had very high withdrawal rates. Another important limitation was the severe gastrointestinal side effects of NSAIDs in these trails. Thus, it is of importance to continue to focus on AD treatment using NSAIDs using a perfective experimental design.

In our previous study, we demonstrated that MSL, as a novel NSAIDs agent, had limited macroscopic effects on gastric mucosa after long-term oral administration in mice (Xin et al., 2014). Moreover, based on the characteristics of inflammatory responses in early stages of AD, we designed a study to elucidate the preventive effect of NSAIDs in an APP/PS1 mouse model. Our results showed that oral administration of MSL at doses of 150 mg/kg and 300 mg/kg for 4 months, improved spatial learning deficits in APP/PS1 mice and ameliorated memory impairment. In addition, MSL-treated mice displayed a better learning capability in finding a hidden platform by reduction of escape latency in a MWM test. In the memory probe trial, APP/PS1 mice that received MSL treatment were much better in searching for the target quadrant and the site where the platform was located when compared to APP/PS1 control mice. The behavioral test confirmed the protective effects of MSL for AD progression *in vivo*, which was consistent with clinical findings of long-term use of NSAIDs against AD incidence (Vlad et al., 2008). Moreover, these findings were also supported FJB immunofluorescence analysis indicating that FJB-positive cells in the cerebral cortex were significantly decreased in MSL-treated APP/PS1 mice. These results implied that MSL may be beneficial to reduce neuroprotective effects by suppressing neuronal degeneration. Ultrastructural changes in neurons were consistent with FJB data. Moreover, TEM analysis further confirmed that MSL treatment prevented the ultrastructural changes, including shrinkage and swelling of neurons and the surrounding neuropil. Based on *in vivo* studies on cognitive function, such as neuronal degeneration and ultrastructural changes, we indicated that MSL treatment was beneficial for the progression of AD pathology in early therapy.

In a previous study, it was demonstrated that mitochondrial dysfunction of neurons was involved in the progression of AD (Michael and O’Banion, 2007). Extensive literature is available, supporting that mitochondrial dysfunction and oxidative damage play a role in the pathogenesis of AD (Onyango, 2018). Moreover, oxidative damage occurs early in the neurons in the AD brain, before the onset of significant Aβ deposition. The current understanding implies that oxidative stress is a hallmark of AD and responsible for activation inflammatory processes (Mhatre et al., 2004). Here, we evaluated the protective effects of MSL on mitochondrial ability and oxidative stress using APPsw cells. The present study revealed that treatment with MSL improved reduced MMP in APPsw cells in the presence of copper. In addition, MSL treatment caused anti-oxidative effects by preventing ROS production in copper-induced APPsw cells. These findings demonstrated that anti-inflammatory treatment could protect neuronal cells by improving mitochondrial ability through increasing the reduced MMP and protecting mitochondria from oxidative stress.

Oxidative stress not only contributes to mitochondrial dysfunction but also activates signaling pathways that alter the processing of APP or tau. Recently, several pathways have been confirmed that showed a relation between oxidative stress and AD pathology. For example, oxidative stress increased the expression of β-secretase through activation of c-Jun amino-terminal kinase and p38 MAPK (Schnöder et al., 2016). In most studies, the MAPK signaling pathway was focused on gliacyte, and the data showed that MAPKs are significantly involved in the activation of microglia and astrocytes and the subsequent inflammatory response, leading to neurotoxicity and AD progression. Increasing evidence suggested that pro-inflammatory mediators produced by activated microglia and astrocytes induced neuronal MAPK phosphorylation and cell death in AD (Chang et al., 2010; Ghosh et al., 2013). Here, we focused on neuronal SH-SY5Y cells overexpressing the Swedish mutant form of human APP to investigate MAPK activation affected by MSL treatment. We demonstrated that in APPsw cells, JNK, p38 and ERK1/2 were activated and phosphorylated by copper treatment, and that MSL reduced the increased phosphorylated levels of JNK and p38 MAPK. Thus, our results implied that MSL elicited a neuronal protective effect by inhibiting JNK and p38 MAPK-related inflammatory responses.

In previous studies, it has been demonstrated that NSAIDs reduce the risk of AD in patients with rheumatoid arthritis. NSAIDs generally inhibit the expression of COX-1 and COX-2 to exhibit anti-inflammatory effects (McGeer et al., 1990; Akiyama et al., 2000). Traditionally, neurons were believed to be a passive bystander in neuroinflammatory processes. However, increasing evidence has indicated that neurons themselves can produce inflammatory mediators, such as PGE_2_ and related COX, and other cytokines (Heneka and O’Banion, 2007). Therefore, neurons participate in inflammatory processes and will be disrupted during AD progression. MSL is a novel NSAIDs that non-selectively inhibited the expression of COX-1 and COX-2 to mediate anti-inflammation effects (Xin et al., 2014). Here, we evaluated the protection of MSL treatment on neuronal cells. Our results demonstrated that treatment with MSL protected copper-stimulated APPsw cells by inhibiting the activity of COX-1 and COX-2 and the subsequent production of PGE_2_. Thus, it is suggested that MSL might suppress the inflammatory activity of neurons by inhibiting COX activity, thereby preventing the progression of AD.

## Conclusion

In summary, we provided evidence that MSL, a novel NSAIDs derived from nature products, protected against Aβ neurotoxicity and identified the underlying mechanisms involved. Our findings indicated a clear rescue of cognitive deficits and neurodegeneration in a mouse model of AD after long-term MSL treatment in the early stages of AD. Moreover, the underlying mechanism may involve Aβ toxicity-mediated neuronal inflammation by suppressing mitochondrial dysfunction and oxidative damage, thereby further preventing subsequent activation of JNK and p38 MAPK signaling pathways. Moreover, the anti-inflammatory effects of MSL also involved inhibiting the expression of COX1/2, which are generally overexpressed in neuronal cells. Thus, we assessed the prevention of MSL on AD and the potential mechanisms involved, and concluded that the therapeutic effects of MSL on AD will need to be further confirmed.

## Author Contributions

JL, XM and TZ designed the experiments, performed the experiments, analyzed the results and drafted the manuscript. YW, LW and GS carried out the behavioral measure. CC and MH carried out the cell cultures. JF and JL carried out the immunofluorescence. DZ synthesized the MSL. All authors read and approved the final manuscript.

## Conflict of Interest Statement

The authors declare that the research was conducted in the absence of any commercial or financial relationships that could be construed as a potential conflict of interest. The reviewer JCS and the handling editor declared their shared affiliation.
